# Prevalence of Mental Disorders in a German Kidney Transplant Population: Results of a KTx360°-Substudy

**DOI:** 10.1007/s10880-022-09861-0

**Published:** 2022-02-23

**Authors:** Katrin Birkefeld, Maximilian Bauer-Hohmann, Felix Klewitz, Eva-Marie Kyaw Tha Tun, Uwe Tegtbur, Lars Pape, Lena Schiffer, Mario Schiffer, Martina de Zwaan, Mariel Nöhre

**Affiliations:** 1 Department of Psychosomatic Medicine and Psychotherapy, Hannover Medical School, Carl-Neuberg-Strasse 1, 30625 Hannover, Germany; 2 Project Kidney Transplantation 360° (NTx360°), Hannover Medical School, Hannover, Germany; 3 Institute of Legal Medicine and Forensic Sciences, Charité-Universiätsmedizin, Berlin, Germany; 4 Department of Psychosomatic Medicine and Psychotherapy, University of Göttingen Medical Centre, Göttingen, Germany; 5 Department of Sports Medicine, Hannover Medical School, Hannover, Germany; 6 Department of Pediatric Kidney, Liver and Metabolic Diseases, Hannover Medical School, Hannover, Germany; 7 Department of Pediatrics II, University Hospital of Essen, University of Duisburg-Essen, Essen, Germany; 8 Department of Nephrology and Hypertension, University Hospital Erlangen, Erlangen, Germany; 9 Department of Nephrology and Hypertension, Hannover Medical School, Hannover, Germany

**Keywords:** Mental comorbidity, Kidney transplantation, Renal transplantation, Mental health, Psychotherapeutic treatment

## Abstract

In patients after kidney transplantation (KTx) an increased rate of affective and anxiety disorders has been observed. Repeatedly, a relationship between mental health issues and increased morbidity and mortality in KTx recipients has been reported. However, information on the prevalence of mental disorders in KTx patients is scarce. As part of the structured multimodal follow-up program (KTx360°), mental disorders were examined in 726 patients after KTx through structured diagnostic interviews using the Mini-DIPS Open Access. Overall, 27.5% had a current and 49.2% a lifetime mental disorder. Only 14.5% with a current mental disorder reported to be in treatment. Affected patients were younger, more often female, reported more symptoms of anxiety and depression and less perceived social support. While comparable to the rate in general population samples, the prevalence of mental disorders should attract attention. The low treatment rate requires an improved identification of afflicted patients and provision of specialist treatment.

I*SRCTN registry*, https://doi.org/10.1186/ISRCTN29416382, date of registry: 03.05.2017.

## Introduction

It is well known that compared to the general population, the prevalence of mental disorders is higher in patients with chronic illnesses (Turner & Kelly, 2000). Mental disorders are also very common in patients with chronic kidney disease (CKD) (Cohen et al., 2007; De Sousa et al., 2008; Kimmel et al., 1998; Stasiak et al., 2014). Depression is the most frequently reported psychiatric condition in CKD, especially in those with end-stage renal disease (ESRD) (Palmer et al., 2013). The prevalence of depression among patients with CKD can be as high as 75%, depending on the diagnostic criteria and the studied population (Anvar-Abnavi and Bazargani, 2010). As reported by Kimmel et al. (1998), hospitalizations due to mental disorders (especially depression, anxiety, and substance abuse) are 1.5 to 3 times more common among patients with CKD compared to individuals with other chronic diseases. As a psychosocial evaluation of kidney transplant candidates is not mandatory prior to listing for kidney transplantation in Germany, there is no reliable information available on the prevalence of mental disorders in this particular group of patients.

For patients with ESRD, kidney transplantation (KTx) is the treatment of choice. In comparison to dialysis treatment, KTx is associated with an improved quality of life as well as lower morbidity and mortality (Wyld et al., 2012). In Germany, currently, about 23,000 patients are living with a transplanted kidney (Girndt et al., 2016). Several studies evaluated depression in KTx recipients (Palmer et al., 2013; Shirazian et al., 2017). In most studies, self-rating scales were used to diagnose depression (mean prevalence of 26.6%). However, the gold standard for identifying mental disorders is a diagnostic assessment by a mental health professional, ideally using structured diagnostic interviews (Ali et al., 2016; Bolton, 2001; Shabani et al., 2021). In the systematic review and meta-analysis of Palmer et al. (2013) on the prevalence of depression in chronic kidney disease only three small studies were included in which the prevalence of depression was evaluated in KTx recipients based on diagnostic interviews (Arapaslan et al., 2004; Ceyhun et al., 2010; Merino et al., 2011). The three studies performed in Turkey and Argentina comprised a total of 122 KTx patients, reporting an overall estimated prevalence of 25.7% (CI 12.8–44.9%). Little is known regarding the prevalence of other mental disorders in KTx patients.

Major depression increases the risk for mortality in patients after organ transplantation (Dew et al., 2015). In kidney transplant recipients, major depression has shown to be associated with a higher risk for graft failure (Dew et al., 2015). The mechanisms behind the association between major depression and higher morbidity and mortality are not well understood. However, research suggests that behavioral as well as pathophysiological aspects may explain this connection (Dew et al., 2015). Additionally, associations between mental disorders and nonadherence to the immunosuppressive medication (ISM) have been reported in the past (Belaiche et al., 2017; Denhaerynck et al., 2007). Nonadherence to the ISM can severely affect transplant outcomes and might contribute substantially to transplant failure (Pinsky et al., 2009; Sellares et al., 2012). However, patients with a successfully treated mental disorder and stable social support seem to have a transplant result comparable to patients without a mental disorder (Corbett et al., 2013).

The aims of our study were (1) to evaluate the prevalence and distribution of current and lifetime mental disorders in a large sample of KTx recipients with the support of an expert-rated structured diagnostic interview and compare these results to the prevalence found in German and U.S. population-based samples, (2) to examine the provision of medical care services with respect to the treatment of mental disorders, (3) to investigate putative associations between the presence of mental disorders and socioeconomic, medical, transplant-specific and psychological variables, and (4) to examine the specific impact of the renal disease and the transplantation process on the development of stress-related mental disorders including adjustment disorder and posttraumatic stress disorder.

## Methods

### Sample Selection

The study participants were recruited within the structured post-transplant care program KTx360° (Pape et al., 2017) between May 2017 and July 2019. The project took place in the transplant centers of Hannover Medical School and Hann. Münden in Lower Saxony, Germany.

A mental health professional (specialist for psychosomatic medicine (MD) or psychologist) performed a psychosocial assessment in all patients. The participants were asked to complete several questionnaires. The Institutional Ethics Review Board of Hannover Medical School approved the study (number 3464-2017), and all participants gave written informed consent.

### Instruments

#### Diagnostic Interview

Mental disorders were diagnosed with the help of the diagnostic interview for mental disorders—the Mini-DIPS Open Access (OA) (Margraf and Cwik, 2017). The Mini-DIPS OA is a structured clinical interview for diagnosing mental disorders similar to the SCID. It is applicable for adults and assesses current and lifetime mental disorders according to DSM-5 and ICD-10. The Mini-DIPS OA covers the mental disorders most commonly seen in clinical settings: anxiety disorders, affective disorders, obsessive–compulsive disorders, trauma and stress-related disorders, sleep–wake disorders, disorders related to psychotropic substances and dependent behaviors, and suicidal behavior. Reliability and validity of the instrument have been tested before (Margraf and Cwik, 2017). Following the recommendations, a two-stepped procedure was performed. At first, participants were asked a screening question regarding the specific mental disorder. If the question was answered positively, further diagnostic questions followed to confirm the diagnosis.

#### Medication Adherence Rating Scale (German Version, MARS-D)

To evaluate adherence to the immunosuppressive medication, the German version of the Medication Adherence Rating Scale (MARS-D) was used (Horne & Weinman, 1999; Mahler et al., 2010). The self-report instrument consists of five items and is rated on a 5-point Likert scale leading to a total score between 5 and 25. Higher ratings are indicative of better adherence. Nonadherence was defined by a total score below 25, as suggested by others (Bouwman et al., 2017; Lee et al., 2017). Patients were asked to focus on their immunosuppressive medication when answering the questions exclusively. With the approval of the original authors, we adapted the MARS-D for this purpose. Cronbach's α for the total score was 0.638.

#### Hospital Anxiety and Depression Scale (HADS-D)

Symptoms of psychopathology also were assessed by self-report. The German version of the Hospital Anxiety and Depression Scale (HADS), a self-report instrument, which is specifically designed to evaluate levels of anxiety and depression in physically ill patients, was used to measure anxiety and depression (Herrmann-Lingen & Buss, 1994; Hermann-Lingen et al., 1995; Zigmond & Snaith, 1983). The questionnaire consists of two subscales, "depression" and "anxiety," with seven items each. The items are rated between 0 and 3, leading to a sum score between 0 and 21, with higher results indicating higher levels of depression or anxiety. Cronbach's α was 0.859 for depression and 0.818 for anxiety.

#### Perceived Social Support (F-SozU K7)

Perceived social support was measured using the German F-SozU K-7 (Dunkel et al., 2005; Fydrich et al., 2009). The instrument consists of seven items rated on a 5-point Likert scale, ranging from 1 ("does not apply") to 5 ("exactly applicable"). Thus, a total score between 7 and 35 can be reached, with higher scores indicating higher perceived social support. Cronbach's α in our sample was 0.895.

### Medical, Sociodemographic, and Transplant-Specific Variables

The estimated glomerular filtration rate (eGFR) was extracted from the medical records at the time of the psychosocial assessment. This marker indicates kidney functioning. In kidney transplant recipients, the eGFR is an essential variable to define transplant functioning and to recognize difficulties regarding the transplanted organ, e.g. organ rejection or over-immunosuppression. Sociodemographic and donation-specific variables including sex, age, partnership status, level of education (≥ 12 years/ < 12 years), donation type (living/deceased donor), and time since KTx were acquired using a self-report questionnaire. Missing information was taken from the medical records.

### Statistical Analysis

For each variable descriptive statistics (percentages, means, and standard deviations (SD), medians, and interquartile ranges) were calculated. Mann–Whitney-U tests and Chi-square tests were utilized to calculate differences between two groups (patients with and without current or lifetime mental disorder).

Statistical significance was set at *p* < .05. All statistical analyses were performed using IBM® Statistical Software Package of Social Science (SPSS®, Chicago, IL, USA) version 26.

## Results

### Sample characteristics

Until July 2019, 726 patients at least 16 years of age participated in the psychosocial assessment within the KTx360° trial. Table 1 comprises the demographic and clinical details of the sample. The mean age was 52.6 years; the majority of the participants were male (58.5%). Most participants attended less than 12 years of formal education (74.1%). The mean eGFR was 45.6 ml/min/1.73 m^2^. Time since KTx was on average 64.9 months, and the mean time on dialysis before transplantation was 61.1 months. About a quarter of the patients (28.9%) received their transplants from a living donor. Overall, 39.3% of the participants reported nonadherence regarding the immunosuppressive medication intake (MARS-D < 25).

**Table 1:** Patient characteristics

Patient characteristics	All, *n* = 726
Age (years)	
Mean (SD)	52.6 (14.3)
Median (IQR)	54.8 (20.1)
Gender, *n* (%)	
Female	301 (41.5)
Male	425 (58.5)
≥ 12 years school attendance, *n* (%), *n* = 719	
Yes	186 (25.9)
No	533 (74.1)
Donation type, *n* (%)	
Living	210 (28.9)
Deceased	516 (71.1)
eGFR (ml/min/1.73 m^2^), *n* = 717	
Mean (SD)	45.6 (18.7)
Median (IQR)	42.9 (24.3)
Time since KTx (months)	
Mean (SD)	64.9 (68.9)
Median (IQR)	48.3 (78.9)
Time on dialysis (months), *n* = 712	
Mean (SD)	61.1 (49.5)
Median (IQR)	55.5 (83.0)
MARS-D Score (adherence), *n* = 697	
Mean (SD)	24.4 (1.2)
Median (IQR)	25.0 (1.0)
HADS-D Anxiety score, *n* = 695	
Mean (SD)	5.1 (3.9)
Median (IQR)	4.0 (6.0)
HADS-D Depression score, *n* = 698	
Mean (SD)	4.3 (3.9)
Median (IQR)	3.0 (6.0)
FSozu K7 (sum score), *n* = 698	
Mean (SD)	30.0 (6.0)
Median (IQR)	32.0 (8.0)

*eGFR* Estimated glomerular filtration rate, *F-Sozu K7* Questionnaire for perceived social support, *HADS-D* Hospital Anxiety and Depression Scale, *MARS-D* Medication Adherence Rating Scale, *SD* standard deviation, *IQR* interquartile range

### Prevalence, Distribution and Treatment of Mental Disorders

Information on the prevalence of mental disorders can be found in Tables 2 and 3. In our study, 200 patients (27.5%) were diagnosed with at least one current mental disorder with 9.9% (*n* = 72) meeting the criteria for a mood disorder (e.g. depression, bipolar disorder, dysthymia), 7.7% (*n* = 56) for a neurotic, stress-related, and somatoform disorder (e.g. obsessive–compulsive disorder, posttraumatic stress disorder, adjustment disorder, hypochondriasis, somatization disorder, somatoform autonomic dysfunction, persistent somatoform pain disorder and neurasthenia), and 7.4% (*n* = 54) for an anxiety disorder (e.g. agoraphobia, social phobias, specific phobias, panic disorder, generalized anxiety disorder, mixed anxiety and depressive disorder and unspecified anxiety disorder).

**Table 2:** Current and lifetime prevalence rates of mental disorders according to ICD-10 categories in KTx recipients (n = 726)

	Current *n* (%)	Lifetime *n* (%)	Treatment (combined)	
			Current *n* (% of pats. with current dx)	Total *n* (% of pats. with lifetime dx)
Any mental disorder	200 (27.5)	357 (49.2)	29 (14.5)	122 (34.2)
Number of mental disorders				
0	526 (72.5)	369 (50.8)		
1	142 (19.6)	229 (31.5)		
2	44 (6.1)	83 (11.4)		
≥ 3	14 (1.9)	45 (6.2)		
Any mental and behavioral disorders due to the use of psychotropic substances	41 (5.6)	126 (17.4)	1 (2.4)	34 (27.0)
Alcohol	2 (0.3)	16 (2.2)		
Opioids	1 (0.1)	1 (0.1)		
Cannabinoids	1 (0.1)	6 (0.8)		
Sedatives and hypnotics	2 (0.3)	2 (0.3)		
Cocaine	0	1 (0.1)		
Tobacco	37 (5.1)	117 (16.1)		
Others	1 (0.1)	3 (0.4)		
Any affective disorder	72 (9.9)	170 (23.4)	16 (22.2)	81 (47.6)
Bipolar affective disorder	1 (0.1)	1 (0.1)		
Mild depressive episode	7 (1.0)	11 (1.5)		
Moderate depressive episode	12 (1.7)	20 (2.8)		
Severe depressive episode	2 (0.3)	4 (0.6)		
Depressive episode, unspecified	0	4 (0.6)		
Recurrent depressive episode, mild	11 (1.5)	11 (1.5)		
Recurrent depressive episode, moderate	21 (2.9)	21 (2.9)		
Recurrent depressive episode, severe	2 (0.3)	2 (0.3)		
Recurrent depressive episode, remitted	0	84 (11.6)		
Dysthymia	16 (2.2)	16 (2.2)		
Any anxiety disorder	54 (7.4)	66 (9.1)	10 (18.5)	27 (40.9)
Agoraphobia	14 (1.9)	17 (2.4)		
Social phobias	1 (0.1)	1 (0.1)		
Specific phobias	27 (3.7)	31 (4.3)		
Panic disorder	13 (1.8)	18 (2.5)		
Generalized anxiety disorder	3 (0.4)	3 (0.4)		
Mixed anxiety and depressive disorder	2 (0.3)	3 (0.4)		
Anxiety disorder, unspecified	2 (0.3)	3 (0.4)		
Any neurotic, stress-related, and somatoform disorder	56 (7.7)	100 (13.8)	4 (7.1)	36 (36.0)
Obsessive–compulsive disorder	6 (0.8)	8 (1.1)		
Posttraumatic stress disorder	4 (0.6)	8 (1.2)		
Adjustment disorder	37 (5.1)	71 (9.8)		
Hypochondriasis	3 (0.4)	3 (0.4)		
Somatization disorder	0	1 (0.1)		
Somatoform autonomic dysfunction	1 (0.1)	1 (0.1)		
Persistent somatoform pain disorder	5 (0.7)	5 (0.7)		
Neurasthenia	0	3 (0.4)		
Any eating disorder	6 (0.8)	8 (1.1)	0	1 (12.5)
Anorexia nervosa	1 (0.1)	3 (0.4)		
Binge-eating disorder, grazing	5 (0.7)	5 (0.7)		
Other diagnoses			9 (33.3)	15 (45.5)
Sleeping disorder	20 (2.8)	22 (3.0)		
Personality disorder	2 (0.3)	2 (0.3)		
Impulse control disorder	1 (0.1)	3 (0.4)		
Schizophrenia	2 (0.3)	3 (0.4)		
Other diagnoses	3 (0.4)	4 (0.5)		
Disorders due to psychological or behavioral factors affecting diseases classified elsewhere	9 (1.2)	12 (1.6)	3 (33.3)	6 (50.0)

*dx* diagnosis

**Table 3:** Comparison of prevalence and distribution of mental disorders

	Our data	Jacobi et al. (2016)	NSDUH (2020)
Population	German KTx recipients	German general population	U.S. general population
Reference time	Current mental disorder	Twelve-month prevalence	Twelve-month prevalence
Any mental disorder (including tobacco use)	27.5%	NR	24.5%
Any mental disorder (excluding tobacco use)	24.7%	27.8%	NR
Use of psychotropic substances (excluding tobacco use)	0.7%	5.7%*	
Tobacco use	5.1%	13.2%	
Any affective disorder	9.9%	9.8%	
Any anxiety disorder	7.4%	15.4%	
Obsessive–compulsive disorder	0.8%	3.6%	
Posttraumatic stress disorder	0.6%	2.3%	
Any psychotic disorder	0.3%	2.6%	
Adjustment disorder	5.1%	NR	

*NR* not reported

*Use of illegal substances excluded

Regarding the lifetime prevalence of mental disorders, 49.2% (*n* = 357) had been suffering from a mental disorder at some point in their life; 23.4% (*n* = 170) from a mood disorder, 17.4% (*n* = 126) from a mental and behavioral disorder due to the use of psychotropic substances (including tobacco), and 13.8% (*n* = 100) from a neurotic, stress-related, or somatoform disorder. Excluding tobacco use, 24.7% of the participants were affected by at least one current mental disorder.

Of the 200 patients diagnosed with a current mental disorder, 14.5% (*n* = 29) reported receiving psychotherapeutic or psychopharmacological treatment at the time of evaluation. The percentage of patients with a lifetime mental disorder having received psychotherapeutic or psychopharmacological treatment at some point was 34.2% (*n* = 122).

### Comparison Between Patients With and Without Mental Disorders

Comparison between patients with and without at least one mental disorder can be found in Tables 4 and 5. Patients with a current mental disorder were younger, showed higher values in the anxiety and depression scale of the HADS-D, and reported lower perceived social support measured with the FSozU. In addition, in the group of patients with a current mental disorder, the percentage of women was higher compared to the group of patients without a current mental disorder.

**Table 4:** Comparison of KTx recipients with and without a current mental disorder

Patient characteristic	Patients with current dx (*n* = 200)	Patients without current dx (*n* = 526)	Statistics *X*^2^-test, Mann–Whitney U-test
Age (years)			***Z* = − 2.778, *p* = .005**
Mean (SD)	50.5 (13.3)	53.4 (14.6)	
Median (IQR)	52.0 (18.8)	55.7 (19.2)	
Gender, *n* (%)			***X***^**2**^** = 9.295 (df = 1), *p* = .002**
Female	101 (50.5)	200 (38.0)	
Male	99 (49.5)	326 (62.0)	
≥ 12 years of school attendance, *n* (%)			*X*^2^ = 0.830 (df = 1), *p* = .362
Yes	56 (28.3)	130 (25.0)	
No	142 (71.7)	391 (75.0)	
Donation type, *n* (%)			*X*^2^ = 0.273 (df = 1), *p* = .601
Living	55 (27.5)	155 (29.5)	
Deceased	145 (72.5)	371 (70.5)	
eGFR (ml/min/1.73 m^2^)			*Z* = − 1.257, *p* = .209
Mean (SD)	44.4 (18.4)	46.1 (18.8)	
Median (IQR)	41.8 (24.8)	43.1 (24.1)	
Time since KTx (months)			*Z* = − 0.431, *p* = .666
Mean (SD)	62.2 (67.0)	65.9 (69.7)	
Median (IQR)	42.0 (75.1)	50.8 (80.1)	
Time on dialysis (months)			*Z* = − 0.555, *p* = .579
Mean (SD)	62.6 (49.5)	60.5 (49.6)	
Median (IQR)	59.0 (81.0)	54.5 (82.0)	
MARS-D score (adherence)			*Z* = − 0.305, *p* = .760
Mean (SD)	24.3 (1.5)	24.4 (1.1)	
Median (IQR)	25.0 (1.0)	25.0 (1.0)	
HADS-D anxiety score			***Z* = − 8.994, *p* < .001**
Mean (SD)	7.4 (4.2)	4.2 (3.3)	
Median (IQR)	8.0 (7.0)	4.0 (4.0)	
HADS-D depression score			***Z* = − 8.647, *p* < .001**
Mean (SD)	6.7 (4.7)	3.4 (3.2)	
Median (IQR)	6.0 (8.0)	2.3 (4.0)	
FSozU (sum score)			***Z* = − 5.263, *p* < .001**
Mean (SD)	28.0 (6.7)	30.8 (5.6)	
Median (IQR)	30.0 (12.0)	33.0 (6.0)	

*dx* diagnosis, *eGFR* Estimated glomerular filtration rate, *F-Sozu K7* Questionnaire for perceived social support, *HADS-D* Hospital Anxiety and Depression Scale, *MARS-D* Medication Adherence Rating Scale, *SD* standard deviation

**Table 5:** Comparison of KTx recipients with and without a lifetime mental disorder

Patient characteristic	Patients with lifetime dx (*n* = 357)	Patients without lifetime dx (*n* = 369)	Statistics *X*^2^-test, Mann–Whitney U-test
Age (years)			*Z* = − 1.324, *p* = .186
Mean (SD)	52.0 (13.7)	53.1 (14.8)	
Median (IQR)	54.5 (20.0)	55.0 (20.5)	
Gender, *n* (%)			***X***^**2**^** = 10.978 (df = 1), *p* = .001**
Female	170 (47.6)	131 (35.5)	
Male	187 (52.4)	238 (64.5)	
≥ 12 years school attendance, *n* (%)			*X*^2^ = 0.098 (df = 1), *p* = .754
Yes	90 (25.4)	96 (26.4)	
No	265 (74.6)	268 (73.6)	
Donation type, *n* (%)			*X*^2^ = 0.268 (df = 1), *p* = .593
Living	100 (28.0)	110 (29.8)	
Deceased	257 (72.0)	259 (70.2)	
eGFR (ml/min/1.73 m^2^)			*Z* = − 0.345, *p* = .730
Mean (SD)	45.5 (19.0)	45.8 (18.5)	
Median (IQR)	43.5 (25.6)	42.8 (24.0)	
Time since KTx (months)			*Z* = − 1.313, *p* = .189
Mean (SD)	61.9 (69.2)	67.7 (68.7)	
Median (IQR)	42.4 (74.0)	53.9 (83.9)	
Time on dialysis (months)			*Z* = − 0.272, *p* = .786
Mean (SD)	61.5 (49.4)	60.7 (49.7)	
Median (IQR)	56.5 (86.0)	55.0 (81.0)	
MARS-D Score (adherence)			*Z* = − 0.172, *p* = .863
Mean (SD)	24.4 (1.3)	24.4 (1.2)	
Median (IQR)	25.0 (1.0)	25.0 (1.0)	
HADS-D anxiety score			***Z* = − 7.285, *p* < .001**
Mean (SD)	6.2 (4.1)	4.0 (3.2)	
Median (IQR)	6.0 (7.0)	3.0 (5.0)	
HADS-D depression score			***Z* = − 7.696, *p* < .001**
Mean (SD)	5.6 (4.4)	3.2 (3.0)	
Median (IQR)	5.0 (6.0)	2.0 (4.0)	
FSozU (sum score)			***Z* = − 5.502, *p* < .001**
Mean (SD)	28.7 (6.5)	31.3 (6.5)	
Median (IQR)	31.0 (10.0)	33.0 (5.0)	

*dx* diagnosis, *eGFR* Estimated glomerular filtration rate, *F-Sozu K7* Questionnaire for perceived social support, *HADS-D* Hospital Anxiety and Depression Scale, *MARS-D* Medication Adherence Rating Scale, *SD* standard deviation

In the group of patients with a lifetime history of mental disorders, we found significantly more women compared to the group of patients without a lifetime mental disorder. Above that, patients with a lifetime mental disorder reported more symptoms of depression and anxiety and less perceived social support.

Regarding transplant-specific variables including donation type, eGFR, time since KTx and time on dialysis, no differences were found between the groups.

### Stress-Related Mental Disorders

Regarding posttraumatic stress disorder (ICD-10: F43.1) and adjustment disorder (ICD-10: F43.2), we analyzed the triggering psychosocial stressor. Results can be found in Table 6. Overall, 79 patients were affected by a posttraumatic stress disorder and/or an adjustment disorder. Furthermore, in 39.2% of the patients, experiences concerning the kidney disease (e.g., difficulties in coping with the symptoms or life changes associated with the disease), the treatment (e.g., difficulties with severe side effects), or the transplantation process (e.g., distressing memories associated with the intensive care treatment or complications after transplantation) could be identified as the psychosocial stressor responsible for the development of the stress-related mental disorder.

**Table 6:** Assumed causal factor for stress-related mental disorders

	Causal stressor		
	Related to the transplantation process (*n*)	Related to the nephrological disease/dialysis (*n*)	Related to other incidents (*n*)
Posttraumatic stress disorder (F43.1)	1	–	7
Adjustment disorder (F43.2)	21	9	41
	22 (27.8%)	9 (11.4%)	48 (60.8%)

## Discussion

This study adds to the limited data on the prevalence of mental disorders in KTx recipients. We were able to perform structured diagnostic interviews in a large sample of KTx recipients and identified a current mental disorder in 27.5% (24.7% when excluding tobacco use) and a lifetime mental disorder in 49.2% of the patients. Even though the result that one in four KTx patients is affected by a current mental disorder appears surprisingly high, these numbers are in line with prevalence rates found in the general population:

The most recent study using an interview-based approach in a nationally representative sample of the German population was performed by Jacobi et al., (2014, 2015, 2016). The 5303 participants were between 18 and 79 years old. The Composite International Diagnostic Interview (CIDI) was used to assess symptoms, syndromes, and diagnoses according to DSM-IV, covering 25 diagnoses (Jacobi et al., 2014, 2016). The authors reported a twelve-month prevalence rate of mental disorders of 27.8% (excluding tobacco use), compared to a point prevalence of 24.7% (excluding tobacco use) in our sample of KTx recipients. Regarding the U.S., results from the 2019 National Survey on Drug Use and Health (NSDUH) are available. The study was performed as a household interview survey including 50,731 individuals aged 18 years or older (NSDUH, 2020). The authors found that 24.5% of U.S. adults met criteria for a mental disorder (including tobacco use) in 2019 (NSDUH, 2020).

Regarding the distribution of mental disorder, there are some differences to note: In the study of Jacobi et al., (2014, 2015, 2016), anxiety disorders were the most common mental disorders (15.4%), followed by mood disorders and neurotic, stress-related, and somatoform disorders. In our sample of KTx recipients, mood disorders (9.9%) were most common, followed by neurotic, stress-related, and somatoform disorders and anxiety disorders.

Concerning substance and tobacco use more pronounced differences became apparent: The prevalence of tobacco and substance use was significantly lower in KTx patients compared to the general population sample (Table 3). There are different conceivable explanations: On the one hand, KTx candidates and patients are strongly advised to quit smoking as it might negatively impact graft function and mortality (Agarwal et al., 2011; Nourbala et al., 2011; Opelz and Dohler, 2016; Weinrauch et al., 2018). The rate of tobacco use in our sample of KTx recipients was only about a third of the rate found in the general population sample (5.1% vs. 13.2%) (Jacobi et al., 2014, 2015, 2016). This result is also in line with the findings of Dew et al. (2018), who reported that between 1 and 3% of KTx recipients showed non-adherent behavior to transplant program recommendations regarding substance use (including tobacco use). On the other hand, ending (illegal) substance use and/or alcohol misuse is often required to be considered an eligible transplant candidate and to be listed for transplantation (Kröncke et al., 2018). This condition might lead to low substance use rates in KTx patient samples.

Additionally, it should be kept in mind that severe mental disorders, including psychosis or unstable mental conditions, are often seen as relative contraindications for organ transplantation (Molnar et al., 2018). Several organ transplant societies support this, and even though it is not explicitly stated in the guideline of the German Medical Association, it can be hypothesized that it is common practice in transplant centers in Germany as well (Molnar et al., 2018). The reasoning behind this procedure can be explained by worries regarding a possible reoccurrence of the mental disorder, a potentially higher risk for non-adherent behavior, and the fear that a lack of mental treatment or social support might lead to problems in the post-transplant phase (Butler et al., 2017; Klapheke, 1999; Molnar et al., 2018). However, the data supporting these assumptions are limited and contradictory (Butler et al., 2017; Klapheke, 1999; Molnar et al., 2018). Nevertheless, it is essential to acknowledge that current practice most probably leads to an underrepresentation of patients with these disorders in transplant recipient samples.

As already known from the general population (Jacobi et al., 2014, 2015, 2016; NSDUH, 2020), women and younger individuals were suffering more frequently from mental disorders in our study sample. Interestingly, we found no association between educational level and the prevalence of mental disorders. Several studies suggest that lower social status, often strongly correlated with a lower educational level, is associated with a higher prevalence of mental disorders (Scott et al., 2014). At the same time, others reported that a lower educational level is associated with reduced access to kidney transplantation (Hod & Goldfalb-Rumyantzev, 2014). On that basis, it seems possible that patients with a particularly low education status might be underrepresented in this sample. Therefore, associations between education level and mental disorders as described by others could not be confirmed.

As expected, patients with a current mental disorder reported higher levels of self-reported anxiety and depression measured with the HADS compared to patients not currently affected by a mental disorder. The same can be said about patients with a history of mental disorders compared to patients who have never been affected by a mental disorder. This result is not surprising as the questionnaires are designed to detect symptoms of mental disorders (Herrmann-Lingen & Buss, 1994; Hermann-Lingen et al., 1995; Zigmond & Snaith, 1983).

In the patients without a current or lifetime mental disorder, we found higher perceived social support. Literature suggests that high perceived social support is associated with better mental health (Eom et al., 2013). Some studies even found that high perceived social support might prevent the onset of mental disorders (Kliem et al., 2015). However, in our study, no causal interpretation is possible due to the cross-sectional study design. Additionally, it is important to keep in mind that participants with a mental disorder might perceive social support differently (Eom et al., 2013), which might further emphasize the differences in the results between patients with and without a mental disorder.

In our study, 39.3% of the patients reported non-adherence to the immunosuppressive medication. Even though the percentage appears quite high, it is in line with other non-adherence rates found in the literature (Hugon et al., 2014). Remarkably, we found no difference regarding self-reported adherence in patients with and without a (current or lifetime) mental disorder. In the review of Belaiche et al. (2017), an association between mental disorders (especially anxiety and depression) and lower adherence to the immunosuppressive medication has been described. They also reported an association between the extent of symptoms and the dimension of non-adherence (Belaiche et al., 2017). Further variables often associated with poor adherence are young age, male gender, low social support, unemployment, low educational level, longer time since transplantation, and receiving an organ donation from a living donor (Belaiche et al., 2017). However, in contrast to these other variables associated with poor adherence (Burkhart & Sabaté, 2003), mental disorders are potentially treatable conditions. On this basis, we strongly oppose the unselected exclusion of patients with mental disorders from organ transplantation.

### Treatment

Regarding mental health treatment, 14.5% of the 200 patients diagnosed with a current mental disorder reported receiving psychopharmacological and/or psychotherapeutic treatment. In the group of patients with a lifetime mental disorder, 34.2% had received psychotherapeutic or psychopharmacological treatment at some point in the past. Those numbers are alarmingly low but are comparable to the findings from other studies. In the German general population, (Jacobi et al., 2014), 57% of the participants with a mental disorder during the last twelve months indicated that they had not sought help regarding their mental health (Brandstetter et al., 2017). A significant gap often occurs between the onset of symptoms and the initiation of treatment (Demyttenaere et al., 2004; Thom et al., 2019). On average, patients with anxiety or affective disorders seek treatment six to seven years after the first symptoms appeared (Mack et al., 2014). Moreover, up to three-quarters of the patients with a mental disorder are treated by their general practitioner and do not receive treatment by a mental health professional (Gaebel et al., 2013; Thom et al., 2019). Regarding U.S. adults with a mental illness, 43.8% received treatment in 2019 (NSDUH, 2020). However, an average delay of 11 years between the onset of symptoms and the start of treatment was reported in this sample (NSDUH, 2020; National Alliance on Mental Illness, 2021). Some studies reported a negative impact of (inadequately treated) mental disorders on several outcomes after organ transplantation (Dew et al., 2015; Rosenberger et al., 2012). Mental disorders are treatable conditions and therefore modifiable risk factors for post-transplant outcomes. Successful treatments will likely lead to longer graft functioning and improved health-related quality of life (Mouelhi et al., 2018; Prihodova et al., 2014). Therefore, more attention should be put in improving the low treatment rates by enhancing the diagnostic assessment and patient care for KTx patients and providing low-threshold specialized treatment for mental disorders.

### Stress-Related Mental Disorders

Psychosocial stressors and traumatic events can lead to maladaptive responses when an individual has difficulty adjusting to or coping with a stressful psychosocial event or trauma. Overall, the prevalence rates of current and lifetime adjustment disorders (5.1% and 9.8%, respectively) and posttraumatic stress disorder (0.6% and 1.2%, respectively) were relatively low in our sample. In 40% of the patients, these stress-related disorders were associated with events around the kidney disease, the treatment, or the transplantation process. Bachem et al. (2019) evaluated the frequency of adjustment disorders based on ICD-11 criteria in a sample of organ transplant recipients using a self-rating questionnaire. The 140 participants were recruited through a self-help group, and 10.7% fulfilled the criteria for current adjustment disorder with the disease or its treatment as the initiating stressor. In our sample, the prevalence of adjustment disorder was much lower at 5%, and of those, only about 40% were associated with experiences and events associated with the disease and its treatments. However, the diagnoses in the study of Bachem et al. (2019) were made based on questionnaires. Additionally, the diagnostic criteria have changed between ICD-10 and ICD-11. Above that, it is vital to keep in mind that patients organized in a self-help association might not be representative for the group of organ transplant recipients. This group of patients might feel a higher burden, leading to an overestimation of the prevalence of adjustment disorder. Moreover, adjustment disorder is defined as a transient disorder that remits spontaneously within six months after the triggering event or within two years if there is a persisting triggering factor (Bachem et al., 2019). However, in the study of Bachem et al. (2019), adjustment disorder was diagnosed up to a decade after organ transplantation. In comparison to our study, it is likely that some of these KTx recipients would have been diagnosed with a depressive episode in a structured clinical interview. Above that, it is important to consider that other mental disorders might have been triggered by traumatic events associated with the disease and its treatment, including depressive disorders and anxiety disorders.

Regarding posttraumatic stress disorder (PTSD), a systematic review (Davydow et al., 2015) found that after organ transplantation, the point prevalence of clinician-ascertained PTSD ranged from 1 to 16%. Compared to these results, the prevalence in our sample is relatively low (0.6%). In addition, most studies included in this review were conducted in patients after heart transplantation. It is likely that patients undergoing kidney transplantation receive a less risky operation and spend less time in intensive care units compared to, e.g., patients undergoing heart or lung transplantation (DSO, 2020a, 2020b). Therefore, KTx patients might have a lower risk for developing PTSD associated with events around the kidney disease, the treatment, or the transplantation process.

### Limitations

Even though all eligible participants of the KTx360° trial participating in the psychosomatic evaluation were included in this study, we have to take the possibility of a selection bias into account, as we cannot be sure that the 726 participating KTx patients are representative of the whole population of KTx patients.

In comparison to the population-based samples, we noticed that some diagnoses were underrepresented in our sample. Literature suggests that some mental disorders, including psychosis and mania, are seen as relative contraindications for organ transplantation (Molnar et al., 2018). However, further research seems necessary to examine if this procedure is justified.

It is important to keep in mind that all patients were recruited in German transplant centers. It is well known that the selection of transplant candidates differs between different countries (Steinman et al., 2001). Apart from that, the attitudes regarding seeking mental health treatment vary as well (Krendl & Pescosolido, 2020). These differences may limit the transferability of our results.

In the NSDUH study, the investigators used an interview approach not entirely comparable to our study. This circumstance does not allow comparison of prevalence rates of diagnostic groups.

## Conclusion

In conclusion, in our large sample of KTx recipients, we found a prevalence of mental disorders comparable to German and U.S. general population samples. In contrast to the majority of other studies, the diagnoses were made by a mental health professional with the help of a structured diagnostic interview. Regarding the distribution of diagnoses, minor differences compared to the German general population could be detected. As expected, patients with a mental disorder were younger and more likely to be female. Some mental disorders were directly associated with the transplantation event, the medical treatment, or the nephrological disease.

Alarmingly, only 14.5% of the KTx recipients diagnosed with a current mental disorder received mental health treatment. This finding is worrisome as inadequately treated mental disorders might negatively impact several outcomes in the disease trajectory of organ transplantation (Dew et al., 2015; Rosenberger et al., 2012). However, mental disorders are treatable conditions and therefore modifiable risk factors for post-transplant outcomes. Our results stress the need to increase diagnostic efforts and the patient's access to suitable treatment. At the same time, we have not found any indication that patients with a mental disorder performed worse in transplant-specific outcomes including self-reported adherence to immunosuppressants. Based on our results, we strongly advocate against systematically excluding patients from organ transplantation due to mental disorders.

## Data Availability

The datasets generated for this study are available on request to the corresponding author.
